# 肺结节/肺癌患者全程管理模式的设计与应用

**DOI:** 10.3779/j.issn.1009-3419.2020.103.06

**Published:** 2020-05-20

**Authors:** 丹 刘, 燕 黄, 清华 周, 伦旭 刘, 国卫 车, 铀 卢, 峰 许, 锋 罗, 红利 白, 为民 李

**Affiliations:** 1 610041 成都，四川大学华西医院呼吸与危重症医学科 Department of Respiratory and Critical Care Medicine, West China Hospital, Sichuan University, Chengdu 610041, China; 2 610041 成都，四川大学华西医院健康管理中心 Health Management Center, West China Hospital, Sichuan University, Chengdu 610041, China; 3 610041 成都，四川大学华西医院肺癌中心 Department of Lung Cancer Center, West China Hospital, Sichuan University, Chengdu 610041, China; 4 610041 成都，四川大学华西医院胸外科 Department of Thoracic Surgery, West China Hospital, Sichuan University, Chengdu 610041, China; 5 610041 成都，四川大学华西医院肿瘤科 Department of Thoracic Oncology, West China Hospital, Sichuan University, Chengdu 610041, China; 6 610041 成都，四川大学华西医院放射科 Department of radiology, West China Hospital, Sichuan University, Chengdu 610041, China

**Keywords:** 肺结节, 肺癌早期筛查, 全程管理, Pulmonary nodules, Early screening, Comprehensive management

## Abstract

**背景与目的:**

早期筛查是有效降低肺癌死亡率的重要方式, 然而我国尚缺乏统一、有效的肺癌早期筛查标准与手段。本研究基于四川大学华西医院构建的肺结节/肺癌患者全程管理平台, 将肺结节早期筛查纳入肺癌规范诊疗体系, 以期实现提升肺癌患者生存率。

**方法:**

建立三大研究队列：健康人群筛查队列、肺结节患者队列、肺癌患者队列, 系统、持续收集、分析各队列人群临床诊疗相关大数据。初步计划对肺结节早期筛查效果进行验证。

**结果:**

该平台初步以2015年1月1日-2019年12月31日华西医院40岁以上职工2, 836人作为实施对象, 共诊断66例肺癌, 均经外科手术切除确诊为早期肺癌65例, 1例肺癌伴脑转移。

**结论:**

肺结节/肺癌患者全程管理项目可实现肺结节高效筛查、随访与诊疗, 未来全程管理模式将全面覆盖华西医疗系统, 发挥区域带动作用。

## Abstract (en)

**Background and Objective:**

Mortality of lung cancer can be decreased by early screening effectively. However, consistent and proficient standards & methods have not been established in China. This study was based on pulmonary nodules/lung cancer comprehensive management platform established by West China Hospital, Sichuan University. Early screening of pulmonary nodules was integrated into standard healthcare of lung cancer system, aiming to improve survivals of lung cancer patients.

**Methods:**

Three cohorts were established: healthy populations, pulmonary nodules cohort and lung cancer patients cohort, and related clinical data will be collected and analyzed. Preliminary plan includes verifying effect of pulmonary nodules screening module.

**Results:**

Pulmonary nodules screening was performed in 2, 836 employers (> 40 years old) of West China Hospital. Lung cancers were diagnosed in 66 participants, all receiving surgery to remove the lesions. 65 of them were with early stage diseases, 1 with lung cancer and brain metastasis.

**Conclusion:**

Proficient screening, follow-up and healthcare can be achieved via pulmonary nodules/lung cancer comprehensive management mode, which will be extended all over west China region in future.

原发性支气管肺癌(简称肺癌)始终高居我国癌症发病率、死亡率首位：年新增774, 323例, 年死亡690, 567例^[1]^。我国肺癌年龄标准化发病率虽然与美国、英国等发达国家相近, 但年龄标准化死亡率却是后者的1.4倍^[2]^。肺癌患者总体5年生存率仅为19.8%, 远低于乳腺癌、宫颈癌等恶性肿瘤(5年生存率分别为83.2%和67.6%)^[3]^。我国肺癌患者生存预后差, 关键原因是肺癌早期常无特异性临床症状, 早期诊断困难, Ⅰ期肺癌(早期)我国仅占19.0%, Ⅲ期/Ⅳ期(晚期)占64.6%。由此带来诸多问题, 如治疗效果差, 诊疗不规范、经济负担重, 造成严重的家庭及社会负担^[4]^。

大量的临床研究发现低剂量螺旋计算机断层扫描(low-dose spiral computed tomography, LDCT)用于肺癌筛查, 可发现早期可治愈的肺癌, LDCT可降低20%因肺癌导致的死亡(*P*=0.004)^[5]^。因此, LDCT被世界各国推荐为高危人群肺癌筛查的主要方法, 我国在2010年也启动了基于人群的LDCT肺癌筛查项目^[6]^, 但筛查的高危目标人群仍存在广泛争议。另一方面, 随着胸部CT广泛应用, 肺结节患者检出率明显提高, 但仍缺乏精准的评估手段。虽然肺癌治疗手段不断进步, 但仍存在方案选择随意, 疗程不合理、治疗不规范的问题。因此, 建立基于我国肺癌患者人群特征的高危人群筛查、加强肺结节的规范化管理、实现肺结节的早期精准识别及处理、规范中晚期肺癌患者的全程治疗, 建立肺结节/肺癌患者的多学科全程管理模式, 最终达到提高肺癌患者5年生存率、降低肺癌死亡率的目标。

## 1 对象与方法

### 1.1 研究对象

建立三大研究队列：健康人群筛查队列、肺结节患者队列、肺癌患者队列(图 1)。

**1:**
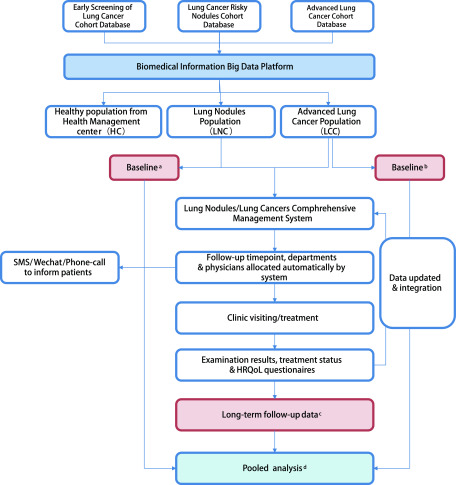
研究设计框架。^a^参考肺结节队列(LNC)人口统计学特征、临床特征、疾病特异性适应症; ^b^参考肺癌队列(LCC)的人口统计学特征、临床特征; ^c^用于合并分析的数据是指随访期间肺结节(LNC)进展, 肺癌队列(LCC)治疗和健康相关生活质量评估; ^d^统计分析。 Framework of study design. ^a^Refer to Lung Nodules Cohort (LNC)-demographic features, clinical features, disease specific indications; ^b^Refer to Lung Cancer Cohort (LCC)-demographic features, clinical features; ^c^Data for pooled analysis refer to Lung Nodules Cohort (LNC)-progression of lung nodules during follow-up, Lung Cancer Cohort (LCC)-therapy and HRQoL; ^d^refer to analysis and statistics.

#### 1.1.1 建立健康人群筛查队列(healthy cohort, HC)

以健康体检人群、社区常驻居民为队列来源, 志愿行低剂量螺旋CT筛查并志愿参加本研究。主要纳入标准：年龄≥40岁且具有以下任一危险因素者：①吸烟≥20包年(或400年支), 或曾经吸烟≥20包年(或400年支), 戒烟时间 < 15年：②有环境或高危职业暴露史(如石棉、铍、铀、氡等接触者); ③合并慢性阻塞性肺疾病、弥漫性肺纤维化或有肺结核病史者; ④既往罹患恶性肿瘤或有肺癌家族史者。

#### 1.1.2 建立肺结节患者随访队列(lung nodule cohort, LNC)

主要纳入标准：①年龄≥40岁; ②志愿参加; ③HRCT发现肺小结节≥4 mm。

#### 1.1.3 建立肺癌患者肺癌随访队列(lung cancer cohort, LCC)

主要纳入标准：①病理确诊肺癌; ②志愿全程管理。

### 1.2 研究设计

该研究为一项前瞻性注册登记研究, 纳入四川大学华西医院肺结节及肺癌患者基本信息、临床诊疗数据及研究相关数据。根据纳入标准, 主诊医师确定患者是否纳入全程管理, 并确定诊疗项目, 包括随访计划、临床检查、诊疗路径及计划, 全程管理中心服务团队通过管理系统平台, 采集患者血液样本及临床数据, 并根据主诊医师诊疗计划, 为肺结节/肺癌患者设定随访时间, 主动分配并预约就诊科室与医师, 预约检查项目、预约入院, 并通过短信、微信平台、电话告知等形式通知患者。由此, 在长期随访中主动实现患者-医师高效对接, 保证患者长期随访依从性以及数据收集的连续性与完整性。

该研究进行过程中, 研究者自全程管理数据库提取指定时间段、相应队列患者数据, 交予审核员审核后进行汇总分析。整个研究实施过程中涉及的数据提取、录入、分析等工作, 均在四川大学华西医院临床试验与生物医学伦理专委会批准下进行。患者签署知情同意, 其个人隐私受严格保护。

### 1.3 观察指标与定义

#### 1.3.1 LNC

① 人口学特征：年龄、性别、民族; ②临床特征：吸烟史、肺癌危险因素情况、合并疾病; ③疾病特异性指标：肺结节数量、位置、大小、容积、类型(实性、部分实性、磨玻璃结节); ④随访期间肺结节进展情况：数量、大小、容积及密度。

#### 1.3.2 LCC

① 人口学特征：同LNC队列; ②临床特征：影像学、肺功能指标、病理类型、临床分期、转移情况、确诊时美国东部肿瘤协作组(Eastern Cooperative Oncology Group, ECOG)评分、敏感基因突变情况; ③治疗情况：外科、放射治疗、化疗、靶向治疗、抗血管生成治疗、免疫治疗及介入(消融)治疗; ④治疗效果：无疾病生存期(disease-free survival, DFS)、复发类型(局部复发、远端转移); ⑤疗效指标：总体生存、无进展生存、客观缓解率[按照实体瘤疗效评价标准(Response Evaluation Criteria in Solid Tumors, RECIST)1.1评估治疗缓解情况]; ⑥治疗安全性：≥3级以上治疗相关不良反应发生率[根据常见不良反应事件评价标准(Common Terminology Criteria for Adverse Events, CTCAE)4.0标准评估不良反应]; ⑦健康相关生活质量评估(health-related quality of life, HRQoL)。

### 1.4 统计学分析

采用SPSS 19.0数据统计包进行统计学分析。计量资料的正态性分布采用*Kolmogorov-Smironov*检验, 符合正态分布的计量资料以均数±标准差(Mean±SD)表示, 非正态分布的计量资料采用中位数(M)及范围表示, 计数资料采用频率或率(%)。符合正态分布的两组比较采用*t*检验。不符合正态分布的两组比较采用*Mann-Whitney U*检验, 两组以上采用*Kruskal-Wallis H*检验。计数资料比较采用χ^2^检验。

## 2 结果

### 2.1 肺结节/肺癌患者全程管理平台建立

四川大学华西医院自主开发的肺结节/肺癌患者全程管理平台(图 2), 以“主动、全程、规范”为管理核心三要素, 通过主动分流、主动干预、主动随访肺结节及肺癌患者, 以落实早期发现、早期治疗、全程管理, 强化三个“规范”——规范筛查、规范随访、规范诊疗, 以期实现肺癌早诊早治及治疗价值的最大化, 降低肺癌患者死亡率。

**2:**
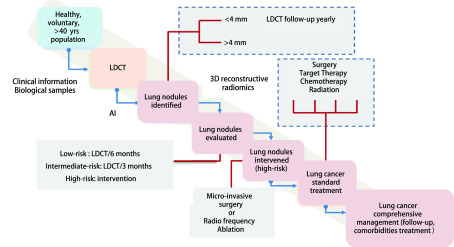
肺结节/肺癌患者全程管理平台流程图。LDCT：低剂量螺旋计算机断层扫描。 Flow chart of pulmonary nodules/lung cancer comprehensive management platform. LDCT: low-dose spiral computed tomography.

**3:**
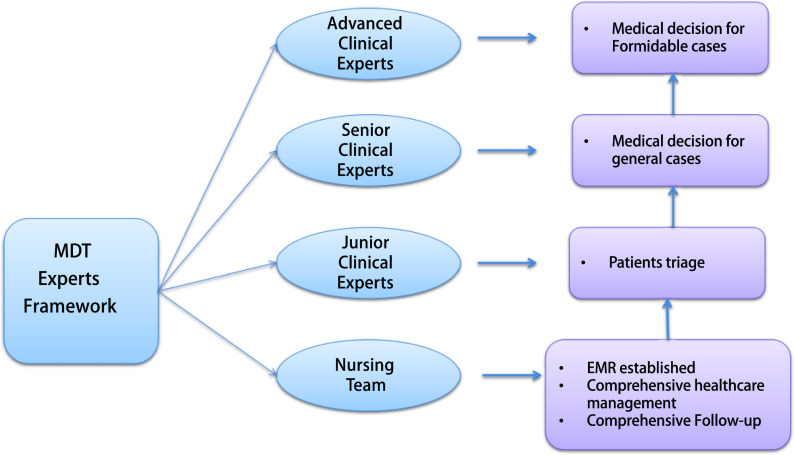
优化、升级的MDT体系：不同层级专家团队。MDT：多学科诊疗。 Optimized and enhanced MDT system: expert teams network. MDT: multi-disciplinary treatment.

### 2.2 肺结节/肺癌多学科团队建立

全程管理聚焦两大核心：其一以早诊早治为核心, 聚焦肺癌高危人群筛查、精准评估肺结节、主动全程管理患者, 以实现肺癌早期诊断、早期治疗, 端口前移。其二以规范全程诊疗为核心, 聚焦肺癌患者诊疗路径, 规范治疗、升级、优化多学科诊疗(multi-disciplinary treatment, MDT)模式, 于MDT体系内部构建不同层级专家/医疗团队, 使各团队在诊疗工作中的职责更为明确、具体, 从而提升中晚期肺癌诊疗的规范性与高效性(图 2)。

### 2.3 华西医院职工全程管理结果

2015年1月1日-2019年12月31日华西医院40岁以上职工2, 836人采用胸部LDCT检查进行肺癌筛查, 其中年龄40岁-94岁, 共诊断66例肺癌(表 1), 均经外科手术切除确诊为早期肺癌65例, 1例肺癌伴脑转移。其中腺癌62例, 鳞癌2例, 腺鳞癌1例, 大细胞癌1例。阳性结节规范随访, 肺癌患者全程管理。

**1:** 体检筛查肺癌分布情况 Distribution of lung cancer patients screened in physical examination

Years	No. examed	No. confirmed (Male) (yr)						No. confirmed (Female) (yr)					Total
		40-59	60-69	70-79	≥80	Sub-total		40-59	60-69	70-79	≥80	Sub-total	
2015	2, 560	0	1	2	1	4		9	2	3	2	16	20
2016	2, 548	0	1	0	1	2		6	1	2	2	11	13
2017	2, 626	3	0	1	1	5		4	1	1	0	6	11
2018	2, 626	3	0	1	1	5		4	1	1	0	6	11
2019	2, 695	2	2	1	2	7		2	3	0	0	5	12
Total	13, 265	9	4	5	5	23		25	7	6	5	43	66

## 3 讨论

从肺癌筛查早期发现肺结节作为切入点, 构建以患者为中心的诊疗体系, 打造全方位、高效覆盖肺癌各疾病阶段的全程管理模式。项目特设全病程管理办公室, 锚定肺癌早期筛查与管理关键节点, 对不同风险层级肺结节患者进行主动分流, 并进行全程随访管理, 破冰实践困局, 保证肺癌早期筛查效率与价值。项目以肺癌诊治面临的重要难点, 提出关键解决对策, 通过全程管理项目, 建立前瞻性肺结节/肺癌患者临床研究队列, 为肺癌早诊早治创新研究奠定基础, 为患者管理模式探索提出新方向。

### 3.1 高危人群的重新定义

欧美与我国相继制定并发布肺癌筛查相关指南、共识, 从评估方法、技术路线、流程管理等方面给出了详尽的推荐建议^[7-12]^, 但高危人群仍存在争议。我国肺癌患者流行病学特征与欧美国家不同, 项目组前期研究发现, 四川地区肺癌患者40岁以上占80%, 约50%肺癌患者为非吸烟人群^[13, 14]^。因此, 华西项目组前期对40岁以上健康人群采用LDCT筛查肺癌研究, 结果发现LDCT在40岁以上体检人群、肺癌高危人群及高危吸烟人群中肺癌检出率分别为0.51%、1.21%和2.01%。进一步对15, 996例健康人群采用低剂量螺旋CT肺癌筛查研究, 结果检出148例肺癌, 按照美国指南纳入高危人群筛查将漏检92.4%的肺癌, 按照中国指南纳入高危人群筛查将漏检78.8%的肺癌, 如按照40岁为高危人群在女性漏检率仅1.45%, 男性漏检率2.74%。首次创新性提出40岁以上的人群应该做低剂量螺旋CT进行肺癌筛查, 基于前期研究结果, 华西全程管理项目以40岁以上所有人群纳入项目。

### 3.2 早诊早治、全程规范管理是降低肺癌患者死亡率的关键环节

随着LDCT肺癌高危人群的筛查肺结节的检出逐年增多, 但该人群的后续诊疗与临床管理面临着诸多实践方面的问题。首先, 由于专业信息的不对称, 肺结节检出患者往往无法对检查结果做出合理解读, 可能误将结节检出等同于肺癌而陷入不必要的焦虑, 或是无视检查结果而错失早期诊断与干预时机^[15]^。除此之外, 肺结节检出患者长期随访依从性有待提升。一项加拿大回顾性研究指出, 肺结节检出患者随访脱落率高达30.2%;对于检出后未进行介入或外科手术的患者, 脱落率则更高(44%)^[16]^。由此可见, 肺结节检出后医务人员及时跟进、医疗系统的及时对接, 以及长期、有效的随访管理是桥接肺癌早期筛查、规范治疗与降低肺癌死亡率的重要环节。

一项美国大型回顾性队列研究显示, 相比早期肺癌患者, 中晚期肺癌不规范治疗(未依照临床指南)比例更高, 且规范治疗患者5年生存率显著更高^[17]^。因此, 全程管理项目对肺结节及肺癌患者MDT模式进行优化探索, 独创分层MDT体系, 明确各层级专家、医务人员工作职责, 大幅提升肺癌患者诊疗规范度。

### 3.3 人工智能、影像组学、介入呼吸诊疗新技术等助力肺癌患者早期诊治

肺结节的精准识别, 依赖于对肺结节的定量定性分析。影像数据爆炸式增长与人工诊断力量严重不足的矛盾更加突出, 亟需高自动化、高诊断效率与高诊断精确度的影像人工智能工具协助肺结节的精准诊疗及管理。研究发现, 影像人工智能能够协助肺结节精准识别及诊断, 敏感性和特异性能够达到94.4%和95.5%。“影像组学”通过使用自动化的数据特征提取算法将影像数据转化为可挖掘的影像特征数据。新型介入呼吸病学诊疗技术, 如导航、超声、机器人等高级支气管镜、CT引导下经皮肺穿刺等呼吸介入手段, 不仅提高了小结节及微小结节诊断率, 也是部分患者的治疗手段。因此, 华西医院全程管理项目将融合影像组学、影像人工智能及新型介入呼吸诊疗技术, 对肺癌高危人群筛查出的肺结节进行肺癌风险度评估及规范管理, 实现肺结节的早期精准识别, 提高肺癌早诊率, 减少过度治疗。随着项目的推进, 该研究将重点关注肺癌疾病演进关键靶点, 明确早期干预的有效方式, 并通过长期随访评估多学科早期精准干预的临床价值, 探索新型患者管理模式, 达到降低肺患者死亡率, 提高5年生存率的最终目标。
